# Paternal and maternal alcohol abuse and offspring mental distress in the general population: the Nord-Trøndelag health study

**DOI:** 10.1186/1471-2458-12-448

**Published:** 2012-06-18

**Authors:** Kamilla Rognmo, Fartein Ask Torvik, Helga Ask, Espen Røysamb, Kristian Tambs

**Affiliations:** 1 Division of Mental Health, Norwegian Institute of Public Health, PO BOX 4404, Nydalen, N-0403, Oslo, Norway; 2 Department of Psychology, University of Oslo, PO BOX 1094, Blinderen, 0317, Oslo, Norway

**Keywords:** Maternal/paternal alcohol abuse, Mental distress, Families, Anxiety/depression, Population study

## Abstract

**Background:**

The degree to which parental alcohol abuse is a risk factor for offspring mental distress is unclear, due to conflicting results of previous research. The inconsistencies in previous findings may be related to sample characteristics and lack of control of confounding or moderating factors. One such factor may be the gender of the abusing parent. Also, other factors, such as parental mental health, divorce, adolescent social network, school functioning or self-esteem, may impact the outcome. This study examines the impact of maternal and paternal alcohol abuse on adolescent mental distress, including potentially confounding, mediating or moderating effects of various variables.

**Methods:**

Data from the Nord-Trøndelag Health Study (HUNT), a Norwegian population based health survey, from 4012 offspring and their parents were analyzed. Parental alcohol abuse was measured by numerical consumption indicators and CAGE, whereas offspring mental distress was measured by SCL-5, an abbreviated instrument tapping symptoms of anxiety and depression. Statistical method was analysis of variance.

**Results:**

Maternal alcohol abuse was related to offspring mental distress, whereas no effect could be shown of paternal alcohol abuse. Effects of maternal alcohol abuse was partly mediated by parental mental distress, offspring social network and school functioning. However, all effects were relatively small.

**Conclusions:**

The results indicate graver consequences for offspring of alcohol abusing mothers compared to offspring of alcohol abusing fathers. However, small effect sizes suggest that adolescent offspring of alcohol abusing parents in general manage quite well.

## Background

A lot of children have parents who abuse alcohol. It has been estimated that 8.3% of all Norwegian children live in homes where at least one of the parents suffer from an alcohol use disorder [1]. Research indicates a variety of adverse effects of parental alcohol abuse on the offspring. Particularly, offspring of alcohol abusing parents experience externalizing problems more often than offspring of non-abusing parents [2-4]. The existing literature concerning the impact on internalizing problems – such as mental distress - show less consistent findings, as some studies report detrimental effects of parental alcohol abuse [e.g. [5], whereas others do not [e.g. [6]. The inconsistency in the literature and the vast amount of children at potential risk warrants more research on the relationship between parental alcohol abuse and offspring internalizing problems.

The gender of the alcohol abusing parent may impact the outcome of the offspring. Previous results suggest that paternal alcohol abuse may represent the strongest risk factor for externalizing problems, whereas maternal alcohol abuse is more strongly associated with internalizing problems [7,8]. In general, maternal alcohol abuse may lead to more serious psychopathology among the offspring than paternal alcohol abuse [8-10]. Findings regarding paternal alcohol abuse and offspring internalizing problems are somewhat conflicting, but the majority of studies do not report significant impact of paternal alcohol abuse after adjusting for other influential variables [7,9,11]. Several studies do not distinguish between maternal and paternal alcohol abuse, and these studies tend to find an association between parental alcohol abuse and internalizing problems in the offspring [3,5,12-14]. The effects of the combined measures of maternal and paternal alcohol abuse may account for some of the inconsistencies in the literature.

Maternal and paternal alcohol abuse may also bestow different risks based on the gender of the offspring, as some studies find that alcohol abuse in the same-sex parent may have a greater impact on the offspring than abuse in the opposite-sex parent [15,16]. Another study found female offspring of alcohol abusing mothers to be prone to depression, whereas male offspring were prone to anxiety [7]. Other studies have been unable to find differential effects for male and female offspring [12]. Parent–child gender interaction effects have been investigated in a limited number of small studies, and the results need to be interpreted with caution.

Most previous studies have been based on small clinical or community samples. The observed associations may be specific to the sample in which they are investigated and may differ systematically from associations in population based samples. Only a few population studies have investigated the association between parental alcohol abuse and offspring mental health. A Danish registry based study by Christoffersen and Soothill [10] investigated the relative risk of hospital admissions for mental illness for offspring of alcohol abusing parents, compared to the remaining population. Parental alcohol abuse did not significantly predict hospital admissions, after adjustments of other adverse life experiences. A population based study by Kessler and colleagues [17] found a significant effect of parental substance abuse on offspring mental illness. However, none of these studies segregated the risk of developing externalizing and internalizing disorders, which makes it difficult to know if there is an augmented risk specific to internalizing disorders among offspring of alcohol abusers.

The association between parental alcohol abuse and offspring internalizing problems may be confounded, mediated or moderated by a variety of factors that may complicate attributing poor mental health in offspring specifically to the alcohol abuse of the parent. Many of the studies on adverse effects of parental alcohol abuse have not taken into account the confounding or mediating effect of other potentially influential variables. Comorbid parental mental health problems could be a particularly important mediator, which often has been ignored [18]. This may have caused overestimations of the relationship.

Despite the extensive literature, there remain several uncertainties regarding the relationship between parental alcohol abuse and offspring internalizing problems, largely due to lack of control of other influential factors and possibly due to sample characteristics. In the present population based study we want to distinguish the effect of maternal and paternal alcohol abuse and examine whether offspring of alcohol abusing mothers and fathers show more symptoms of mental distress, compared to offspring of non-abusing parents. The confounding, mediating or moderating role of various parental and adolescent factors will be investigated. The design does not permit safe conclusions regarding the status of some of the predictors - as confounders or mediators. Some factors, like age, are confounders by nature, as they are established prior to the onset of alcohol abuse. Other factors may be assumed both to precede and succeed parental alcohol abuse in a causal chain and may thus both confound and mediate the relationship. Nonetheless, we will *a priori* classify the covariates as confounding or mediating factors, keeping in mind that their statuses as confounder or mediator are only tentative.

## Methods

### Sample

The study is based on questionnaire data from the second wave of the Nord-Trøndelag Health Study (HUNT 2 and YoungHUNT), which is a large health screening study of the adult and adolescent population of Nord-Trøndelag county, Norway. The data collections were carried out between 1995 and 1997. All adolescents in Nord-Trøndelag county aged 13 to 19 were invited to participate in YoungHUNT, whereas all inhabitants aged 20 or more were invited to participate in the adult HUNT 2 study. YoungHUNT consisted of a health examination and one questionnaire. Data were collected during school hours in all junior high schools and high schools in the county. The adult HUNT 2-study consisted of a health examination and two questionnaires. The first, Q1, was sent by mail together with the invitation letter and returned at the site of the health examination. The second questionnaire, Q2, was distributed during the health examination, completed at home and returned by prepaid mail. The invitation letter explained that the data would be used both to assess each respondents’ health and in medical research, given that the respondent gave their informed consent [19]. In total, 8984 (91%) adolescents participated in YoungHUNT. Of the 92,936 adults invited to HUNT 2, 65,220 (70.2%) returned Q1, and 57,211 (61.5%) returned both Q1 and Q2. Respondents needed to return both Q1 and Q2 to be eligible for inclusion in our analyses. The adult and adolescent populations were examined at approximately the same time in each of the 24 municipalities in the county. Data on family relationships from public registries were matched to the HUNT 2 and YoungHUNT data by Statistics Norway. These data permitted an identification of who were parents of whom. Only offspring with complete data from both parents were retained in the majority of the analyses, leaving 4012 offspring–parents triads. These will be referred to as the nuclear sample from hereon. Based on the identification number of the mother, 3191 did not have siblings participating in the study, whereas 821 had one or more participating siblings. Relatively few divorced families had complete data. Therefore, supplementary analyses were run segregating paternal and maternal data, including families with only one participating parent, increasing the sample size and the proportion of divorced families in the analyses. In these analyses there were 4778 father-offspring dyads and 5989 mother-offspring dyads.

### Ethics

The manuscript has been approved by the Regional Ethics Committee and the study has been performed in accordance with the ethical standards laid down in the 1964 Helsinki declaration. All respondents gave their written consent for their data to be used for research purposes. The invitation letter explained in detail the implications of giving an informed consent [19].

### Adolescent measures

*Mental distress* was measured by SCL-5, a short version of the Hopkins Symptom Checklist-25 [20] consisting of 5 items, of which two measure symptoms of anxiety and three symptoms of depression (α = 0.79). The short version correlates 0.92 with the original SCL-25 [21]. Responses were given on four-point scales, ranging from “not at all” to “extremely bothered or distressed”. The SCL-5 scores had a highly skewed distribution and were logarithm (ln) transformed to approximate a normal distribution. In order to show effect sizes in terms of fractions of standard deviations, the ln-tranformed SCL-5 scores were standardized.

*Self-esteem* was measured by a short version of the Rosenberg Self-Esteem Scale (RSES) [22] consisting of four statements (α = 0.74). The short version has been found to correlate 0.95 with the original scale [23]. Responses were given on a four-point scale, ranging from “totally agree” to “totally disagree”. The scores were summed and the sum score standardized. Like the other predictor variables, RSES was categorized for the purpose of examining moderator effects. In cases of significant interaction effects, the effects of parental alcohol abuse would be assessed separately in each stratum and compared. RSES scores were categorized into three groups, low, normal, and high self-esteem.

*Social network* was measured by four items (α = 0.47), phrased as follows: *“Have you had someone you have considered to be your best friend during your time at school?”* (No/Yes), *“Do you ever feel lonely”* (“very frequently” to “very rarely/never”), *“How many friends do you have?”* (“None” to “Four or more”), *“Do you feel like you have enough friends”* (No/Yes). The four items were standardized, summed and categorized into four categories, ranging from poor to good social network.

*Personality.* A short version of the Eysenck Personality Questionnaire (EPQ) [24] measured the personality factors extraversion, neuroticism, and psychoticism. Neuroticism was excluded from the analyses due to the content overlap with the outcome variable. The correlation between the original and the short version have been observed to be 0.90 for extraversion and 0.82 for psychoticism [23]. Each factor was measured by six items, with response categories “Yes” or “No”. Separate summative scores were computed for each factor. The Cronbach alpha for extraversion was 0.60 and for psychoticism 0.34. The personality factors were used as continuous measures in the main analyses and dichotomized measures in the moderator analyses. Both were dichotomized at the median.

*School functioning* was measured by 14 items that were factor analyzed, applying an oblique rotation. Choosing four factors gave a good and easily interpretable solution, with factors labelled ‘academic problems’ (five items, α = 0.67, highest loading item: *I have difficulties concentrating in class*), ‘conduct problems’ (four items, α = 0.64, highest loading item: *I get reprimanded by the teacher*), ‘being bullied’ (two items, α = 0.19, highest loading item: *I get bullied by other pupils*), and ‘dissatisfaction at school’ (three items, α = 0.53, highest loading item: *I look forward to going to school*). The measures have previously been used in several HUNT-based studies [2,25,26]. Four response categories ranged from “never” to “very often”. The four factors were used as continuous measures in the main analyses and as dichotomized measures in the moderator analyses. Academic problems, conduct problems and dissatisfaction at school were approximately normally distributed and were dichotomized at the median. Being bullied was skewedly distributed, accordingly the top 15% were categorized as being bullied and the remaining 85% as not being bullied.

*Witnessed parents drunk.* One item asked whether the adolescent had seen his/her parents drunk. The wording of the item did not discriminate between having seen the mother or father drunk. Response categories ranged from “never” to “several times per week” and were collapsed into three categories, “never”, “a few times per year”, or “a few times per month or week”.

*Demography.* The adolescents were asked how many siblings they were living with – coded “none” or “one or more”. Age was grouped into three categories, “12-14”, “>14-16.5” and “>16.5-19” years of age. The offspring were asked if their parents were divorced (“No”/“Yes”).

### Parental measures

*Alcohol consumption (items included in Q1) and alcohol related problems (items included in Q2).* First, the respondent was asked to indicate whether or not he/she was abstaining from drinking alcohol (Yes/No). Alcohol *consumption* frequency was reported as number of times the respondent typically had been drinking alcohol within a one-month period. Amount of consumption was measured as responses to three separate items on beer, wine and liquor, respectively. The sum of reported units of beer, wine and liquor that was typically consumed during a two week period was then summed with reported typical number of occasions of drinking during a month. For instance a person having reported two units of beer, one unit of wine and one unit of liquor, and having reported drinking on average 3 times a months, is scored 7. Alcohol *related problems* were measured by the CAGE Alcohol Screening Questionnaire [27]. Abstaining respondents were instructed to skip CAGE. The mothers and fathers were categorized as alcohol abusers or non-abusers. Paternal alcohol abuse was defined as having scored 20 or more on the consumption index and minimum one positive CAGE response. Maternal alcohol abuse was defined as having scored 13 or more on the consumption index and minimum one positive CAGE response. This definition rendered 169 children (4.2%) with alcohol abusing fathers and 83 children (2.1%) with alcohol abusing mothers. These prevalences are clearly lower than usually reported for alcohol abuse and dependence [28], indicating that the large majority of our cases are true, and mostly severe, cases.

*Parental mental distress (Q1).* Symptoms of anxiety and depression were measured by a slightly modified version of the Hospital Anxiety and Depression Scale (HADS) [29] – consisting of 13 items, of which six measured anxiety and seven depression. The items had four response categories, ranging from “not present” to “highly present”. HADS was supplemented by the CONOR Mental Distress Index (CMD), consisting of seven items, of which three measured anxiety and four depression. CMD is described in detail elsewhere [30]. Each item had four response categories ranging from “no” to “very much”. The item scores from HADS and CMD were summed into a global mental distress index, of which the Cronbach’s alpha was 0.91 for mothers and 0.90 for fathers. The index was standardized and used as a continuous measure in the main analyses and as a dichotomous measure in the moderator analyses, in which the top 10% of the fathers and mothers were classified as being mentally distressed.

*Demographics (Q1).* Parental education was scored as one of four categories ranging from primary school to four years or more at college/university. Information on income was provided by public registry data from the income tax authorities. For our analyses we used family income, scored as standardized average income registered in 1990 and 1995.

### Treatment of missing values

SPSS Missing Value Analysis (MVA), Expectation Maximization (EM) was used to impute values for respondents with valid data for minimum 50% of the alcohol consumption items, SCL-5, social network, and HADS/CONOR. The reductions in missing values for all variables were estimated from all fathers and mothers who returned Q2. Missing values were reduced from 2.8% to 1.6% for SCL-5, from 3.2% to 1.1% for social network, from 11.9% to 0.8% for paternal HADS/CONOR, and from 14.8% to 0.5% for maternal HADS/CONOR. Prior to imputations of the alcohol consumption items, the abstaining respondents, having not completed the alcohol consumption items, were scored zero on all the items. In cases with combined blank consumption responses and valid consumption responses higher than zero, we assumed blank responses to signify no consumption and replaced the missing values with zero. For instance if a person had reported five units of beer and seven units of wine and had left units of liquor open, this was assumed to mean “no liquor”. This reduced missing values for the alcohol consumption items for fathers from 38.1% to 7.9% and for mothers from 47.0% to 11.3%. Subsequently, the alcohol consumption items were imputed for the respondents with valid data for minimum 50% of items, which reduced missing values from 7.9% to 6.3% for fathers and from 11.3% to 9.1% for mothers. Missing values on self-esteem (initially 4.6% missing), extraversion (10.8% missing), psychoticism (9.1% missing) and school functioning (8.4% missing) were imputed for all adolescents with missing responses, resulting in no missing values on these variables.

As most of the analyses included data from three subjects, and only a single missing value for one of the subjects would exclude the whole triad, even further operations serving to reduce missing values were necessary. The scores of the 4.2% with missing values on “having seen parents drunk” were replaced with “never” if the parents reported abstaining from alcohol or drinking less than average, else with “a few times a year”. Missing values on social network were replaced by the most frequent response, “quite good social network”.

### Statistical analyses

Multivariate ANOVA (SPSS, Generalized Linear Models (GLM), Generalized Estimating Equations (GEE)) was conducted to investigate the impact of parental alcohol abuse on the mental distress of the offspring, specifying adolescent SCL-5 as the outcome variable. GEE was applied to adjust for lack of statistical independence between data of siblings. The GLM produces a Wald type III test as the parametric statistical test, giving chi-square statistics. First, analyses were run on the nuclear sample of 4012 offspring, giving crude effects of paternal and maternal alcohol abuse on adolescent mental distress. Next, the effect of maternal and paternal alcohol abuse was adjusted by various combinations of variables. *A priori* the covariates were tentatively classified either as likely confounders or likely mediators. The first analyses included the following confounders: adolescent age, gender, the other parent’s alcohol abuse, parental income and education, having siblings, extraversion, and psychoticism. First, adjustments were made block-wise, with 1) adolescent age, gender and the other parent’s alcohol abuse as control variables together with each of the other two blocks, 2) income, maternal and paternal education, siblings or 3) extraversion and psychoticism, consecutively. Second, all the potentially confounding factors were adjusted for simultaneously. Next, the same procedures of block-wise and full adjustments were made for the following potentially mediating variables: 1) parental mental distress, 2) divorce, 3) adolescent social network, 4)witnessed parents drunk, 5) academic problems, conduct problems, being bullied, dissatisfaction at school and 6) self-esteem. Adolescent age, gender and the other parent’s alcohol abuse were controlled for in all the steps. Ultimately, all the mediating and confounding variables were entered into the model simultaneously, giving a completely adjusted effect of maternal and paternal alcohol abuse. The crude and fully adjusted main effects of all the potential confounders and mediators were also reported.

**Figure 1:**
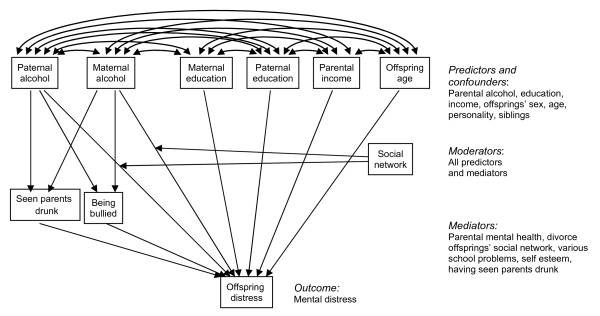
Causal model for the effect of parental alcohol abuse on offspring mental distress, including covariates.

The strength of the associations between paternal or maternal alcohol abuse and offspring mental distress may vary with the adolescent and parental variables mentioned above. Thus, interaction terms between all the aforementioned variables and paternal and maternal alcohol abuse were specified, while controlling for age and gender of the offspring and the other parent’s alcohol abuse. The continuous variables were dichotomized or trichotomized for the purpose of the moderator analyses. Figure 1 shows the causal model, although simplified by only including a sample of confounding, mediating and moderating variables.

Registry data on divorce and remarriage made possible identification of families in which the husband of the mother and, in a few cases, the wife of the father, was not the biological parent of the adolescent. However, these families were not numerous enough to yield reliable information about effects from step-parents. Data from step-parents were excluded from the analyses. A low number of observations of complete data from both divorced parents did not permit reliable assessment of divorce as a confounder or mediator of parental alcohol abuse. In order to better utilize a fuller data set and estimate the effect of divorce, supplementary analyses were run with data from only one of the parents at the time, including data sets in which data from only one of the parents were available.

## Results

### Descriptive statistics

Mean age of offspring was 16.0, of mothers 43.0, and of fathers 45.7. The mean alcohol consumption (summed scale of units of alcohol drunk within a two-week period) for fathers was 4.49 (range 0–67, SD = 4.92), in which approximately 2/3 reported drinking less than the average. The top 15% scored 8 or more. For mothers the mean alcohol consumption was 2.48 (range 0–35, SD = 3.05). The distribution below the mean was similar to the distribution of paternal consumption. The top 15% reported to drink 5 or more units within a two-week period. The point-biserial correlation between parental mental distress and alcohol abuse (informative on the extent to which the effect of parental mental health could confound the association between parental alcohol abuse and mental distress in children) was 0.07 for mothers and 0.08 for fathers.

### Unadjusted and adjusted main effects of maternal and paternal alcohol abuse

The crude effect of paternal alcohol abuse was not significant (see Table 1). Offspring of alcohol abusing mothers had on average 1/3 of a standard deviation (*b* = 0.34) higher scores on SCL-5 than offspring of non-abusing mothers.

**Table 1:** Block-wise and full adjustments of maternal and paternal alcohol abuse on offspring mental distress. N = 4012

	***Paternal alcohol***			***Maternal alcohol***		
	***B***	***CI***	***p***	***B***	***CI***	***p***
***Unadjusted effect***	.12	-.01 - .25	.07	.34	.16 - .53	.00
***Adjusted for blocks of confounders***						
Block 1:						
Age	.12	-.01 - .24	.06	.28	.09 - .47	.00
Gender						
Other parent’s alcohol abuse						
Block 1+2:						
Income	.12	-.00 - .25	.06	.29	.10 - .47	.00
Maternal education						
Paternal education						
Siblings						
Block 1+3:						
Extraversion	.12	.00 - .25	.05	.27	.08 - .46	.01
Psychoticism						
***Adjusted for all confounders***						
Block 1 + 2 + 3	.13	.00 - .25	.05	.27	.09 -.46	.00
***Adjusted for blocks of mediators***						
Block 1+4:						
Mother’s Mental Distress	.10	-.03 - .23	.12	.25	.07 - .44	.01
Father’s Mental Distress						
Block 1+5:						
Divorce	.11	-.01 - .23	.08	.28	.09 - .47	.00
Block 1+6:						
Social Network	.16	.05 - .28	.01	.21	.04 - .38	.01
Block 1+7:						
Seen parents drunk	.10	-.03 - .22	.12	.26	.07 - .45	.01
Block 1+8:						
Academic Problems	.09	-.03 - .20	.14	.24	.07 - .41	.01
Conduct Problems						
Being Bullied						
Dissatisfied at School						
Block 1+9:						
Self-esteem	.13	.02 - .25	.03	.26	.09 - .43	.00
***Adjusted for all mediators***						
Block 1 + 4 + 5 + 6 + 7 + 8 + 9	.11	.00 - .22	.05	.17	.02 - .32	.03
***Adjusted for all confounders and mediators***						
Block 1 + 2 + 3 + 4 + 5 + 6 + 7 + 8 + 9	.08	-.03 - .19	.14	.16	.01 - .31	.03

Paternal alcohol abuse remained an insignificant predictor of offspring mental distress after adjusting for the potentially confounding effect of age, gender, maternal alcohol abuse, income, education and having more than one child. Adjusting for the personality of the offspring and for all the potential confounders in the full model resulted in a significant effect, despite a minimal change in the estimate from *b* = 0.12 to *b* = 0.13. The effect of maternal abuse was reduced from 0.34 to 0.28 after adjusting for the effect of age, gender and paternal alcohol abuse. Adjusting for income, education and having more than one child in one block and for offspring personality in another, did not notably affect the estimates (*b* = 0.29 and *b* = 0.27, respectively), and neither did adjusting for all the potentially confounding variables simultaneously (*b* = 0.27).

The lower part of Table 1 shows the effects of parental alcohol abuse after adjusting for what was *a priori* classified as mediator variables, alongside the variables in block 1. The effect of paternal abuse varied somewhat with type of mediator adjusted for, but entering all predictors considered to be mediators only reduced the direct effect of paternal abuse from 0.12 to 0.11, a just significant value. Effects of maternal abuse were left almost unchanged by entering each separate block of mediators, except for social network, which reduced the effect to *b* = 0.21. Adjusting for all the potentially mediating variables simultaneously, however, reduced the effect of maternal alcohol abuse considerably (*b* = 0.17), but the effect remained significant. To the extent that these variables are real mediators, adjusting for them represents an over-adjustment (removing real, but indirect effects of parental alcohol abuse mediated by factors like impaired social network). Rather, the decrease of the estimate for maternal alcohol abuse from 0.28 to 0.17 tells about the part of the maternal alcohol effects which is mediated by these variables. When entering all the potentially mediating and confounding factors simultaneously into the model, the effect of paternal alcohol abuse was reduced to 0.08, whereas the effect of maternal alcohol abuse was reduced to 0.16. Despite the reduction in effect size, maternal alcohol abuse still significantly predicted offspring mental distress (*p* = 0.03).

Table 2 shows the crude and fully adjusted main effects of both the predictor, confounder and mediator variables on offspring mental distress. Of all the parental predictor variables, maternal alcohol abuse produced the largest group difference. Factors reported by and directly related to the offspring were stronger predictors of offspring mental distress than were factors reported by the parents. The social network of the offspring produced the largest group difference, both unadjusted and adjusted in the full model.

**Table 2:** Crude and fully adjusted effects of all variables on offspring mental distress

	***N***	***B (CI) Unadjusted***	***p.***	***B (CI) Adjusted***	***p.***
***Age***			.00		.00
12-14 years	671	-.37 (−.45- -.29)	.00	-.16 (−.24 - -.08)	.00
>14-16.5 years	1770	-.20 (−.2 - -.14)	.00	-.10 (−.16 - -.04)	.01
>16.5-19 years	1571	0^a^		0^a^	
***Gender child***			.00		.00
Boys	1997	-.45 (−.51- -.39)	.00	-.32 (−.37 - -.26)	.00
Girls	2015	0^a^		0^a^	
***Paternal alcohol***			.07		.14
Father abuses	169	.12 (−.01 - .25)	.07	.08 (−.03 - .19)	.14
alcohol					
Father does not	3843	0^a^		0^a^	
abuse alcohol					
***Maternal alcohol***			.00		.03
Mother abuses	83	.34 (.16 - .53)	.00	.16 (.01 - .31)	.03
alcohol					
Mother does not	3929	0^a^		0^a^	
abuse alcohol					
***Divorce***			.00		.23
Divorced	317	.20 (.08 - .32)	.00	.06 (−.04 - .16)	.23
Not divorced	3695	0^a^			
***Education father***			.01		.01
Elementary and secondary school	890	-.04 (−.12 - .05)	.40	-.08(−.17 - .00)	.05
High school	2152	.08 (.00 - .15)	.05	.03 (−.04 - .09)	.43
University	970	0^a^		0^a^	
***Education mother***			.57		.56
Elementary and secondary school	837	.02 (−.07 - .11)	.71	-.01 (−.09 - .08)	.84
High school	2283	.04 (−.04 - .12)	.30	-.03 (−.10 - .03)	.34
University	892	0^a^		0^a^	
***Income parents***^***b***^		-.01 (−.05 - .02)	.39	.03 (.00 - .07)	.04
***Siblings***			.00		.36
No siblings	1004	.17 (.10 - .24)	.00	.03 (−.03 - .09)	.36
One or more siblings	3008	0^a^		0^a^	
***Father Mental Distress***^***b***^		.10 (.07 - .13)	.00	.04 (.02 - .07)	.01
***Mother Mental Distress***^***b***^		.10 (.07 - .13)	.00	.02 (−.00 - .05)	.08
***Witnessed parents drunk***			.00		.17
Never	3051	-.17 (−.30 - -.03)	.02	.03 (−.09 - .14)	.68
A few times per year	755	-.01 (−.16 - .14)	.91	.08 (−.04 - .21)	.20
A few times per month or week	206	0^a-^		0^a^	
***Social Network***			.00		.00
Poor social network	443	1.08 (.97 – 1.18)	.00	.70 (.57 – .77)	.00
Quite poor social network	545	.70 (.62 - .79)	.00	.47 (.38 - .55)	.00
Quite good social network	1008	.40 (.33 - .46)	.00	.22 (.16- .28)	.00
Good social network	2016	0^a^		0^a^	
***Extraversion***^***b***^		-.12 (−.15 - -.09)	.00	-.03 (−.06 - -.01)	.02
***Psychoticism***^***b***^		.06 (.03 - .09)	.00	.01 (−.02 - .03)	.75
***Academic Problems***^***b***^		.38 (.35 - .42)	.00	.22 (.19 - .26)	.00
***Conduct Problems***^***b***^		.06 (.03 - .10)	.00	.08 (.05 - .11)	.00
***Being Bullied***^***b***^		.16 (.12 - .19)	.00	.05 (.02 - .08)	.00
***Dissatisfied at School***^***b***^		.13 (.10 - .16)	.00	-.06 (−.03 - -.09)	.00
***Self-esteem***^***b***^		-.41 (−.44 - -.39)	.00	-.23 (−.26 - -.20)	.00

^a^ Reference group ^b^ Estimates are standardized beta coefficients.

In order to better assess possible effects of parental divorce, separate analyses were run for all families with complete paternal data (including those without maternal data) and for families with complete maternal data (including those without paternal data). These samples include a larger number of divorced families. The unadjusted main effect of paternal alcohol abuse was significant (N_total_ = 4778, N_alcohol_ = 217, *b* = 0.13, *CI* = 0.00-0.25, *p* = 0.04), as was the unadjusted effect of maternal alcohol abuse (N_total_ = 5989,N_alcohol_ = 131, *b* = 0.31, *CI* = 0.14-0.49, *p* = 0.00). The adjusted estimates in this sample changed only slightly from the estimates in the sample with complete data from mothers and fathers (Paternal alcohol abuse: N = 217, *b* = 0.10, *CI* = −0.01-0.20, *p* = 0.07. Maternal alcohol abuse: *N* = 131, *b* = 0.18, *CI* = 0.05-0.31, *p* = 0.01). The main effect of divorce on offspring mental distress was non-significant both in the nuclear sample and in the segregated analyses (Paternal-offspring data: *b* = 0.04, *CI* = −0.04-0.12, *p* = 0.29. Maternal-offspring data: *b* = 0.05, *CI* = −0.01-0.11, *p* = 0.08).

### Interaction effects

Interaction terms between maternal and paternal alcohol abuse and the parental and adolescent variables were specified. Due to multiple testing, a Bonferroni correction (β = α/n, where n is the number of tests) was applied to the .05 p-level. We performed 38 tests, giving a Bonferroni correction of the alpha level of β = 0.05/38 = 0.001. This criterion rendered no significant interaction effects.

## Discussion

The main aim of the study was to assess the degree to which maternal and paternal alcohol abuse is related to mental distress of the offspring, using a population based sample. Secondly, we wanted to examine whether the relationship was confounded, mediated or moderated by other parental or adolescent factors. Previous research is inconsistent regarding the strength of such a relationship.

### Paternal alcohol abuse and offspring mental distress

The results of the present study showed only a marginal effect of paternal alcohol abuse on mental distress of the offspring. The effect of paternal alcohol abuse hovered around the p-value of .05 – depending on which covariates were included.

### Maternal alcohol abuse and mental distress of offspring

In line with previous research [11], maternal alcohol abuse was found to significantly predict mental distress of the offspring. The effect sizes found in this study are comparable to estimates found in other studies [e.g. [7,11]. The results are also consistent with previous research in reporting graver consequences for offspring of alcohol abusing mothers compared to offspring of alcohol abusing fathers [8-10]. To the extent that the gender specific effect estimates reflect a systematic difference between maternal and parental effects, it may well suggest higher vulnerability to maternal than paternal abuse because mothers tend to occupy a more important caregiver role than do fathers. Studies have shown that the family functioning is negatively affected by maternal alcohol abuse, through causing a less cohesive and organized family environment [31], and poor parenting has been shown to mediate the relationship between maternal and paternal alcohol abuse and child internalizing problems [32]. Another conceivable explanation may be that maternal alcohol abuse is a relatively rare condition, which may lead to more social stigma or worse social consequences for the offspring. Indeed, the social network of the offspring seems to partly mediate the effect of maternal abuse, indicating that having an alcohol abusing mother may make it difficult to get close friends, which in turn causes internalizing problems. The results are supported by previous studies having shown lower social competence among daughters in early childhood [33] and impaired peer relations among young adult children of mothers abusing alcohol [34].

One mediator model investigated the role of parental mental distress in relation to maternal alcohol abuse and offspring mental distress. Previous research is inconsistent regarding the role of parental mental health, as some studies have been unable to find an effect of parental alcohol abuse after controlling for the parent’s mental health [6], whereas others have pointed towards a unique effect of alcohol abuse [3,35]. Given the results of previous research and the known genetic transmission of risk of mental health problems we expected more effect to be removed once adjusting for parental mental distress than actually observed (from 0.28 to 0.25).

There were no significant interaction effects between maternal or paternal alcohol abuse and offspring gender in the present study. Perhaps using separate anxiety and depression measures would have produced different results, in line with research by Corte and colleagues [7], who reported elevated levels of depression among female offspring of alcohol abusing mothers and of anxiety among the male offspring. As our mental distress outcome measures consisted of only five items, differentiating between anxiety and depression was difficult.

The small effects of maternal alcohol abuse and the lack of significant effects of paternal alcohol abuse suggest that most offspring of alcohol abusing parents seem to manage quite well, despite a potentially traumatizing family environment. This may be explained by unmeasured factors such as coping, resilience or protective factors in the surrounding environment of the offspring. Carle and Chassin (2004) found substantial subgroups of children of alcohol abusers to be resilient. Resilience was in turn related to lower levels of internalizing problems [36]. The degree to which offspring experience internalizing problems may vary with complex interfamiliar factors – such as family cohesion, family organization and social support by other family members. Future studies would benefit from including measures such as these, possibly exploring the underlying mechanisms of the relationship.

### Methodological considerations

The existing studies on parental alcohol abuse and offspring mental distress are largely based on clinical samples or community samples of limited generalizability. To our knowledge, the relationship has not previously been investigated in a population based sample. Many earlier studies have been unable to differentiate between maternal and paternal alcohol abuse, and adjusting for mental distress in both parents. The present study is based on data from self-report questionnaires for both parents and offspring, whereas previous research frequently has needed to rely on parental reports of offspring mental distress. In this way our study is much less susceptible to confounding from reporting bias than were previous studies.

However, there are methodological limitations to our study. The adolescent participation rate close to 90% does not represent a problem, but the response rate for the adult sample (61.5%) may have caused a selection bias. However, a study by Torvik et al. [37] investigated non-response in the adult HUNT 2 sample and showed that high alcohol consumption in a previous HUNT study only predicted non-participation in HUNT 2 moderately well (*OR* = 1.27 for the top 3% consumption).

Neither the parent nor the offspring data included diagnosed mental disorders, and short versions of validated scales may provide less valid measures of psychological constructs. However, the short forms have been validated against the original scales, with satisfactory results. The lack of diagnostic measures may have led to misclassifications of parents as alcohol abusers or non-abusers, and although the results were in line with findings from several other studies, limited reliability for the categorizations may have downward biased the effect sizes. The internal consistency of the short form measures of the personality factors extraversion (α = 0.60) and psychoticism (α = 0.34) was lower than expected. Particularly the Cronbach alpha of psychoticism may represent a problem, although the quite high correlations between the short form scales and full scales (0.90 and 0.82) clearly show that internal consistency measures underrates the reliability of these short form scales. Nonetheless our own and previously reported results [38,39], suggest that the internal consistency of the personality dimension psychoticism is far from perfect.

The reported alcohol consumption was lower than expected. The results from the attrition study by Torvik et al. strongly suggest that the low reported alcohol consumption is not primarily due to selection bias. The most likely alternative explanation is substantial under-reporting, which complicates the dichotomization into alcohol abusers and non-abusers. In non-diagnostic alcohol abuse research it is common practice to pre-define alcohol abuse as the top five to 15 percentile or a percentile corresponding to previous prevalence estimates in the same population. In our case that would have resulted in unrealistically low cut-off values. For the same reason, the standard CAGE cut-off of two positive responses was not possible in combination with the alcohol consumption measure. Rather, one positive CAGE response was considered indicative of alcohol abuse in combination with high alcohol consumption. The distribution of the consumption in our sample corresponds well with distributions normally observed in alcohol consumption research, in which approximately 2/3 of the population reports drinking less than the average consumption and 15% reports drinking more than twice that of the average [40]. This suggests that an underreporting has taken place which tends to be proportional with actual consumption, but naturally that does not prevent a certain amount of misclassification. Contaminating the relatively small case group with a certain number of false positives would affect the results much more than would contaminating the large non-case group with a corresponding number of false negatives. Therefore the choice of criteria defining only 4.2% of men and 2.1% of women is methodically sound, but it implies a risk of inflating the effect of alcohol consumption, as the case group may consist of more severe cases than what is usually defined as abusers. Such possible inflation has probably been more than counteracted by deflation due to misclassification of cases and imperfect reliability of the outcome measure. Our measure of parental alcohol abuse is also not perfectly clear-cut regarding type of drinking pattern, which in our abusers may vary from one drink relatively often to binge drinking at some occasions. The extent to which the parents’ drinking is stressful for the children may vary with parental drinking pattern, but we were not able to distinguish between frequent and more irregular but uncontrolled drinking. However, since the criteria for abuse includes at least one positive CAGE score, persons classified as abusers hardly consider their drinking completely problem-free.

The frequently observed comorbidity between alcohol use disorders and anxiety and depression may reflect effects of genes common to alcohol consumption and mental distress [41]. This may induce a heightened risk of mental distress in offspring of alcohol abusers, even in the absence of parental comorbidity, because the relationship may be explained by common genes rather than environment. A Norwegian twin study [42], using measures of mental distress and alcohol consumption similar to ours, found that a moderate phenotypic correlation between alcohol use and mental distress could be fully explained by genes coding for both alcohol use and mental distress among men, and partly explained by common genes among women. The heritability for both phenotypes was approximately 0.5, implying a statistical relationship between parental alcohol abuse and mental distress in the offspring solely on the basis of genes shared by parents and offspring and common to both phenotypes. However, with the low correlation between paternal alcohol abuse and distress observed in our data, it may be shown from calculations based on path analysis that genes alone would only account for a correlation of 0.03 between paternal alcohol consumption and mental distress in offspring. A corresponding spurious genetic effect from mother to offspring is expected to be even lower. In conclusion a substantial confounding from genes is not likely.

## Conclusions

The results of the present study show that mental distress in offspring is statistically associated with - and probably resulting from - maternal alcohol abuse. The evidence of a corresponding effect from paternal alcohol abuse is less clear. The low effect sizes and the limited precision of the estimates do not warrant safe conclusions about a difference between a maternal and paternal effect, however, the trend observed in our data - a stronger maternal than paternal effect - is consistent with previous results. The small effect sizes also suggest that the impact of parental alcohol abuse is probably of limited practical significance for mental health in most, but not all offspring. For those children who develop mental distress due to parental alcohol abuse, some of the effect seems to be mediated through poor social network and school functioning. The moderate effects of parental abuse on adolescent mental health do not mean that parental alcohol abuse is harmless. Many children undoubtedly suffer severely from their parents’ alcohol abuse - sometimes or most of the time. Rather the results probably reflect the extent to which most people, including adolescents, are capable of adjusting to a difficult and potentially traumatizing life situation. Future research would benefit from including measures of resilience and coping as well as protective factors in the family environment, in order to study the underlying mechanisms of the relationship.

As most previous research is based on clinical or community samples of limited generalizability, more population based research on the relationship between parental alcohol abuse and mental distress of offspring is needed. Alcohol abuse is one of the most highly prevalent mental disorders, and only a small fraction of the people affected seeks treatment. We need more knowledge on children of parents with alcohol problems, especially those not seen in the clinics or in the health care system.

## Competing interest

The authors declare that they have no competing interests.

## Authors’ contributions

KR was responsible for the design, carried out the statistical analyses and drafted the manuscript. FAT and HA contributed to the analyses and revision of the manuscript. ER contributed to the design and revision of the manuscript. KT contributed by designing parts of the questionnaires, acquiring data, designing the study, methodological supervision and revising and drafting the manuscript. All authors read and approved the final manuscript.

## Pre-publication history

The pre-publication history for this paper can be accessed here:

http://www.biomedcentral.com/1471-2458/12/448/prepub
